# A novel simulation competition format as an effective instructional tool in post-graduate medical education

**DOI:** 10.1186/s41077-018-0075-4

**Published:** 2018-08-09

**Authors:** Pier Luigi Ingrassia, Jeffrey Michael Franc, Luca Carenzo

**Affiliations:** 1 SIMNOVA - Centro Interdipartimentale di Didattica Innovativa e di Simulazione in Medicina e Professioni Sanitarie, Università del Piemonte Orientale, Via Lanino 1, 28100 Novara, Italy; 2 Department of Emergency Medicine, University of Alberta, 790 University Terrace Building, 8303 - 112 Street, Edmonton, AB T6G 2T4 Canada; 3 SIMNOVA - Centro Interdipartimentale di Didattica Innovativa e di Simulazione in Medicina e Professioni Sanitarie, Università del Piemonte Orientale, Via Lanino 1, 28100 Novara, Italy

**Keywords:** Medical simulation, Simulation competition, SIMCUP, Residents, Trainees, SimWars, Post-graduate education

## Abstract

**Objective:**

Medical simulation competitions are a growing reality. This study aims at exploring if a novel format of simulation competition (SIMCUP) can be an effective educational format in post-graduate education.

**Design:**

We designed a 2-day event that included scientific educational lectures, an orientation to the competition, familiarization with the simulation lab, and competition time. Day 1 was devoted to preliminary rounds and was structured using an Objective Structured Clinical Examination (OSCE)-like system. On day 2, the first four teams advanced to semi-finals and then to finals, which were held using a classical SimWars style.

**Setting and subjects:**

A total of 14 four-participant teams participated in the event over two editions (Ed.1 in 2015 and Ed.2 in 2016).

**Interventions:**

External referees evaluated both technical and non-technical skills for each simulated scenario. Each participant was also administered pre- and post-test questionnaires covering self-perception about the confidence in managing simulated clinical cases, educational effectiveness, satisfaction with the simulation experience, and previous simulation training.

**Main results:**

Overall participants found SIMCUP a useful learning experience, rating it 10 [9, 10] and 10 [7.75–10] out of 10 for Ed.1 and Ed.2, respectively. Participants reported, using a 10-point semantic differential scale ranging from “1 - strongly disagree.” to “10 - strongly agree,” finding both days to be educationally effective: day 1 was rated 9 [7–10] and 9 [8–10] as day 2 was rated 8 [7–10] and 8 [7–10] for Ed. 1 and Ed. 2, respectively.

Participants’ self-perception regarding the confidence of managing the specific scenarios significantly improved immediately after the event as measured by pre- and post-questionnaires for all stations and during both editions.

**Conclusion:**

This study suggests that simulation competition can serve as an effective instructional format in residency training.

**Electronic supplementary material:**

The online version of this article (10.1186/s41077-018-0075-4) contains supplementary material, which is available to authorized users.

## Background

The benefits of simulation-based training in residents’ education have been recently well described [1–5]. It has been demonstrated that simulation can meet the general educational goals of transfer of knowledge, strengthening of cognitive strategies [6], and skill development [7] while adding a dimension of team training [8]. By focusing on adult learning theories, simulation offers its learners deliberate practice and experiential learning [9]. Moreover, training that employs simulation technologies could serve as an important adjunct to learning in the setting of reduced practical exposure due to the reduction in resident work hours globally [10–12]. Excellence in professional development is dependent on time available to practice, motivation, and perseverance [13].

An important change in resident medical education is the arrival of millennial students. To ensure success, medical educators need to know and accept the unique characteristics of these new learners: they often prefer to work in groups with hands-on experiences, enjoying trial and error [14]; they frequently expect learning to be interactive, creative, and fun; they often enjoy thinking laterally [15]. The use of gamification is becoming more and more popular to motivate teaching and learning, also in the medical field [16]. Gamification is the process by which users are encouraged and enticed to perform tasks by incorporating elements of game design and competition. Inherent reward and enjoyment can foster motivation. The effectiveness of competition in medical education has been well supported in the literature [17–19]. It has also been documented that competition can boost residents’ engagement in simulation training [20].

Onstage competitions, called SimWars, are now very popular in multiple specialties. In the SimWars, two teams perform the same scenario to the jury and the audience. Either the referee panel or an audience vote determines which team moves on to the next round of competition [21]. SimWars have been demonstrated to be effective for resident training and helpful for professional development [22, 23]. Taking inspiration from the SimWars, we modified the competition format and designed a simulation competition for residents with the aim of engaging participants to partake in deliberate practice and to experiment using different types of simulations and simulators. We postulated that simulation competition can have an educational value and not only be a mere competitive game. The manuscript describes our 2-year experience with SIMCUP (SIMULATION CUP) and its grounding pedagogical and educational rationales. The objective of this study is to present the new format of simulation competition and to investigate whether the new format was perceived, by the participants, to be an effective educational experience.

## Methods

The study took place at the SIMNOVA Simulation Center based at the Università del Piemonte Orientale in Novara, Italy. The study was submitted to the local ethics committee, which reviewed it and awarded an exemption letter (prot. 713/CE). Each participant was informed about data collection and signed a consent form about audio-video and data collection. This manuscript was prepared following the recommendations of the reporting guidelines for healthcare simulation research [24].

### Competition design

The competition was designed as a 2-day event that included scientific educational lectures, an orientation to the competition, familiarization with the simulation lab, and competition time. Day 1 was devoted to preliminary rounds and was structured using an Objective Structured Clinical Examination (OSCE)-like system [25]. The first day included six simulation stations in which each team was expected to perform a variety of clinical tasks within a given time period (10 min). Evaluation was performed using predetermined criteria formulated to demonstrate competency of skills and attitudes. A seventh station in the form of a technical skill fair gave the participants the opportunity to test their own abilities and manual skills in different areas. At the end of day 1, the four highest ranking teams moved on to the semifinals. On Day 2, semi-finals and finals were held using a classical SimWars style. The semi-finalists and finalists performed their simulation in front of the audience consisting of those teams who did not qualify for the finals, the faculty, and the judges. To keep the final simulations secret, the semifinals and final teams were confined to a room away from the main simulation stage. A detailed description of day 1 and day 2 stations is presented in Table 1.

**Table 1:** Title, type, and simulator used and scenarios played at each station for both editions of SIMCUP

Station	Title	Type	Edition	Simulator	Scenario
Day 1					
1	Medical adult	High fidelity	2015	Gaumard HAL S3201	Acute pulmonary edema
			2016		Acute upper GI bleeding
2	Advanced cardiac life support	Medium fidelity	2015	Laerdal ALS Simulator	Cardiac arrest following hyperkalemia
			2016		
3	Disaster triage	Virtual reality	2015	e-semble XVR	Triage of ten casualties following motor vehicle accident
			2016		Triage of ten casualties following train accident
4	Pediatric/neonatal	High fidelity	2015	Laerdal SimBaby	Birth asphyxia
			2016		Dehydration and hypoglicemia in a small children
5	Obstetric emergency	High fidelity	2015	Gaumard Noelle S57x.100	Eclampsia
			2016		Post-partum hemorrhage
6	Pre-hospital trauma	High fidelity	2015	Gaumard HAL S3101	Traumatic lower limb amputation with shock
			2016	Standardized Patient	Open fracture of the lower limb with active bleeding from femoral artery
7a	Emergency bronchoscopy	Skill station	2015	Simbionix Bronch Mentor	Removal of a foreign body
			2016	Airway Ldt Orsim	
7b	Lumbar puncture	Skill station	2015	Kyoto Kagaku Lumbar Puncture Simulator II	Lumbar puncture execution
			2016		
7c	FAST ultrasound	Skill station	2015	Standardized patient	FAST execution
			2016	3DSystem U/S Mentor	
7d	Basic surgical skill	Skill station	2015	Basic Surgical Instruments and SimuLab Tissue Standard Model	Suture execution
			2016		
7e	Ultrasound guided CVC placement	Skill station	2015	SimuLab Central Line System	Placement of central venous line under US guidance
			2016		
7f	Quality CPR	Skill station	2015	Laerdal QCPR ResusciAnne	Achievement of perfection in chest compression rate and timing
	Chest drain placement		2016	SimuLab Trauma Man System	Chest drain placement
Day 2					
Semi-finals	Adult	High fidelity	2015	Gaumard HAL S3201	Traumatic brain injury
	Obstetric emergency		2016	Gaumand Noelle S57x.100	Domestic violence in a 33-week pregnant lady
	Pediatric/neonatal		2016	Laerdal SimBaby	Congenital diaphragmatic hernia
Finals	Obstetric emergency	High fidelity	2015	Gaumard Noelle S57x.100 Laerdal SimBaby	Cardiac arrest with peri-mortem C-section and neonatal resuscitation
	Adult		2016	Gaumard HAL S3201	Acute anaphylaxis with complete airway obstruction and need for surgical airway in an outpatient settings

At the end of each simulation, a debriefing was carried out. Day 1 simulations were followed by facilitator-led individual team debriefing, while during day 2, each final scenario was followed by a plenary critical reflection with a public interaction between the facilitators and the participating teams. Facilitators were the same instructors running each simulation station and final scenario. SIMCUP can be resource intensive especially from the human resource aspect. We planned a ratio of facilitator/instructor to participant around 1:2.5. The facility allowed ten simultaneous simulation sessions to be carried out. Instructors volunteered their time during SIMCUP.

### Population

SIMCUP was open to residents from any Italian residency program and from any level of training. Participants voluntarily signed up for the event. Four members composed each team and multidisciplinary team were encouraged. The first edition of SIMCUP was conducted in 2015 (Ed. 1) and the second one in 2016 (Ed. 2) with the program still going on today.

### Endpoints

This is a descriptive study centered on participant’s satisfaction, participant’s self-perception of effectiveness, and knowledge gain. We used the first two of *Kirkpatrick’s* four levels of evaluation to assess this. Kirkpatrick’s tool is a model for assessing training programs. The first level explores participants’ satisfaction with the training program: overall event quality, satisfaction with the simulation, and effectiveness of the training format. The second expresses the knowledge gain obtained with the educational process measured as difference between pre- and post-event assessment of participant confidence in managing specific critical scenarios [26].

Moreover, technical and non-technical skill performance was quantitatively measured for each simulation scenario.

### Participants self-perception and feedback

On day 1, participants were asked to fill out a paper-based questionnaire about (1) demographics including previous training specifically organized for the simulation competition and (2) baseline self-perceived levels of confidence in managing critical care scenarios. Participants were then invited to fill out a second electronic questionnaire 1 week after the competition. The questionnaire consisted of the following content sections: (1) overall appreciation of the competition, the format, and stressfulness of the simulations; (2) satisfaction with the simulation experience; (3) effectiveness of the training format; and (4) self-perceived levels of confidence in managing critical care cases after the simulation experience.

For the section about satisfaction with the simulation experience, the previously validated Satisfaction with Simulation Experience Scale (SSES, a 5-item Likert scale) was used [27, 28]. For the other sections, responders were asked to reply using a 10-point semantic differential scale ranging from “1 - strongly disagree” to “10 - strongly agree.”

### Performance assessment

Two or three raters at each station judged team performance. The case designers developed a technical skills scoring sheet for each simulation scenario with a predetermined maximum score. These scoring sheets were simple checklists scored as 0 (no), 2 (yes), or 1 (yes, but incomplete). The total sum of each checklist was then transformed in a decimal score ranging from 0 to 1 by means of dividing the achieved score by the maximal theoretical score. Non-technical skills were measured using either the English or the Italian version of the global rating scale (GRS), which includes six items ranging from 0 to 7 with a maximum possible score of 42 [29, 30]. All raters were experts in critical and emergency care and were the same in both editions of the competition. All raters were familiar users of the assessment tools and participated in a pre-event briefing regarding assessment procedure. All measurements composed the final ranking score and served for the competition progression.

### Statistical analysis

Descriptive statistics were summarized using median and percentiles. Pre-post-competition differences in self-perception regarding confidence were tested using the Wilcoxon signed-rank test.

## Results

A total of seven different teams registered to each of the two events. Demographics of the participants are presented in Table 2.

**Table 2:** Demographics of event participants per edition

	Ed.1	Ed.2
	*n* = 28 (%)	*n* = 28 (%)
Age (mean), year	30	30
Female gender	14 (50)	16 (57)
Residency training year		
PGY1	5 (18)	1 (4)
PGY2	8 (29)	10 (36)
PGY3	6 (21)	5 (18)
PGY4	7 (25)	4 (14)
PGY5	2 (7)	8 (29)
Training program		
Anesthesia/critical care	17 (61)	12 (43)
Emergency medicine	7 (25)	9 (33)
Geriatrics	0 (0)	3 (11)
Internal medicine	2 (7)	2 (7)
Pediatrics	0 (0)	1 (4)
Cardiology	2 (7)	1 (4)

*PGY* post-graduate year

### Overall appreciation, satisfaction with simulation, and educational effectiveness

Overall participants found the SIMCUP as a useful learning experience, rating it globally 10 [9, 10] and 10 [7.75–10] out of 10 for Ed.1 and Ed.2, respectively.

The global results for the Satisfaction with Simulation Experience Scale were 4 [3–5] and 5 [4, 5] out of 5 for Ed. 1 and Ed. 2, respectively.

Educational effectiveness of each topic as rated by participants is presented in Table 3. Regarding methodologies, all participants from both years reported finding both days to be educationally effective: the OSCE-like format of day 1 nine [7–10] and 9 [8–10] as well as the SimWars-like format of day 2 eight [7–10] and 8 [7–10] for Ed1 and Ed2, respectively. Participants self-rated their stress level during the simulations as 8 [7–10] for Ed.1 and 8.5 [6–10] out of 10 for Ed.2.

**Table 3:** Educational effectiveness of each simulation scenario/topic as rated by participants using a 10-point unanchored semantic scale. Results presented as median and 25–75 percentile

	Ed.1	Ed.2
High fidelity, medical adult (1)	9 [8–10]	9 [8–10]
Medium fidelity, ACLS (2)	8 [5–10]	9 [7–10]
Virtual reality, disaster triage (3)	6 [3–8]	8 [7–9.25]
High fidelity, pediatric/newborn (4)	9 [5–10]	9.5 [7–10]
High fidelity, obstetrical emergency (5)	8 [6–9]	8 [6.75–10]
High fidelity, pre-hospital trauma (6)	9 [7–10]	9.5 [8–10]

### Participant’s confidence in managing critical scenario

Participants’ self-perception regarding the confidence of managing the specific scenarios significantly improved immediately after the event as measured by pre and post-questionnaires for all stations and during both editions. Global self-perception about the level of confidence in managing the cases improved from 4 [3–5] to 6 [4–7] in Ed.1 (*p* < 0.05) and from 5 [3–6] to 7 [5–8] in Ed. 2 (*p* < 0.05) out of a scale of 10.

**Fig. 1:**
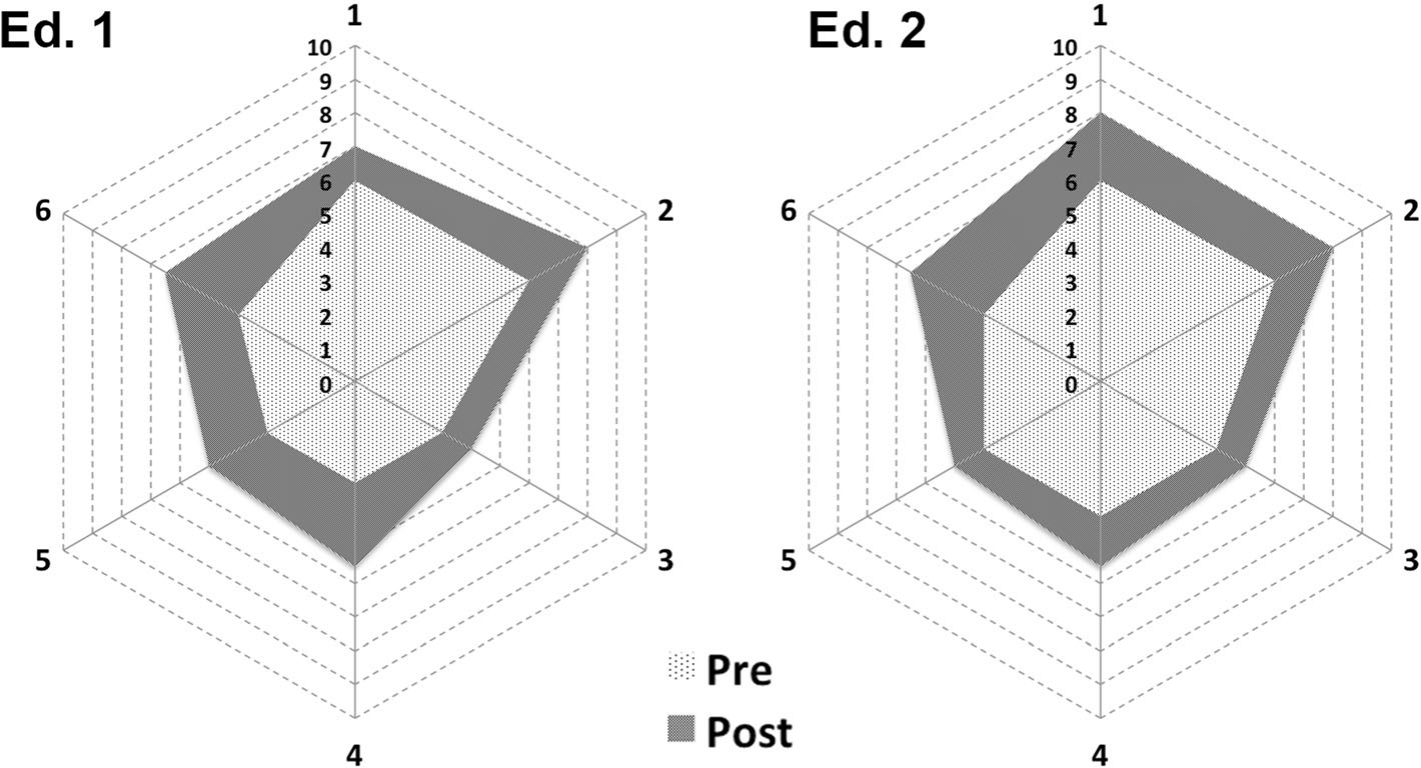
Radar charts presenting median self-confidence perception level variation before (dotted white) and after (gray) the event. Apexes of the polygon represent each simulation station (1 medical adult, 2 advanced cardiac life support, 3 disaster triage, 4 pediatric/neonatal, 5 obstetric emergency, 6 pre-hospital trauma). Axes in each radar chart represent the 10-point Likert scale

Details about each station are presented in the Additional file 1 while the detailed variation between pre- and post-event for each simulation station is represented in Fig. 1.

### Performance assessment

Performance was homogenous between participants of both editions. Details for each station are presented in the Additional file 1. Post hoc visual inspection of team assessment plots (presented in the Additional file 1) between non-technical skills versus technical skills suggests a possible stronger correlation between the two when human factor skills, measured by GRS, are either very high or very low.

### Training

Five teams out of 7 (71%) from Ed.2 reported that they organized some sort of training before attending the event. All five teams reported paper-based study as well as low-fidelity simulation training (BLS Mannequins, homemade simulators), while only 3 teams reported training in a medium to high-fidelity setting. Training data were not collected in Ed. 1.

## Discussion

This study suggests that the proposed simulation competition format is both perceived educationally effective and can improve self-confidence in participating trainees. This suggests it might be a meaningful instructional format to educate residents in clinical management, psychomotor abilities and communication, and teamwork skills.

To our knowledge, the event represents the first training format involving residents from many different Italian residency programs throughout the country, as well as from several different specialties broadly related to critical care. Residents were able to practice their skills and demonstrate abilities in a competitive yet controlled environment, and develop collegiality and teamwork.

The majority of residents reported very high satisfaction both with the event in general as well as with simulation experience measured with the SSES. Although originally developed by nursing researchers, the SSES is generic in nature, allowing its use in other health-related discipline studies. It addresses different aspects of the simulation experience, such as facilitators, debriefing, and reflection, and connects them to clinical reasoning. Residents showed high levels of satisfaction in the SSES, and these results are very important in terms of professional development and clinical performance. Participant satisfaction has been associated with greater involvement in the process and greater motivation for learning [31]. Thus, the participants’ satisfaction may be a good measure to evaluate the teaching and the educational format itself as well [32].

When asked about the level of self-perceived confidence and proficiency before and after participation in the competition, surveyed residents reported a significant improvement in their self-perceived confidence and proficiency. It is known that self-confidence is an important mediating factor that contributes to the extent to which one approaches learning and persists towards achievement of goals and expertise [33]. In addition, previous studies suggest a correlation between experience and self-perceived competence [34–36].

Our findings suggest that a competitive yet controlled simulation environment is a positive learning experience. This is consistent with previous studies, which highlight how the competitive nature of such events provides opportunities for participants to mature in the psychomotor and affective domains of adult learning [17, 19, 37]. Even the competition-induced stress plays as positive role, as stressful situations improve knowledge retention and, up to a certain level, performance [38, 39]. For high-stress professions, such as critical care physicians, training under close-to-real stressful settings helps future practice. Previous literature suggests that stress during competition can have a beneficial effect to participants [23].

The SIMCUP incorporated several learning theories into one event to maximize education. All residents had the opportunity to experience simulated clinical cases in the preliminary rounds of day 1. On day 2, those who were not directly involved in the competition on stage were able to observe the process. This allowed them all to apply their previously acquired competencies in the clinical setting and to learn from both achievement and errors. A number of recently published educational articles expand Kolb’s original work on experiential learning, which stated that learning is the process whereby knowledge is created through the transformation of experience [40].

All SIMCUP participants began by completing a simulated critical event (first phase of Kolb’s cycle). They then came together for structured debriefing: reflective observation and analysis of a concrete experience were carried out through participants’ narrations, questions, and statements with reference to relevant experienced problems and situations that occurred during the simulated event (second phase). These reflections were then assimilated as abstract concepts from which new implications for action were drawn (third phase). These new implications could be actively and immediately experimented and tested in the following simulated event in the circuit (fourth phase).

Observational learning is considered one of the most powerful learning mechanisms [41]. SIMCUP participants were not only able to practice themselves but also watch and observe other team practicing and performing. This is particularly important especially for those teams who did not qualify on day 2 and did not have a chance to be hands-on on the second day. They had however the chance to reflect on other teams’ actions by examining and observing other team simulations and participating in plenary debriefing. SIMCUP combined individual skill assessment with group-learning format. Effective teams work towards a goal by using shared knowledge and skills, aligning with social constructivism theory, which recognizes the value of social interactions in the learning process [42]. The provision of a simulation gives learners a sense of immediacy and involvement where time and the chosen response are critical to successful outcomes [43].

The performance evaluation allowed assessment of technical and non-technical skills that might deserve a corrective intervention. Participants performed poorly in the station related to management of mass casualty events, pointing out a weakness in the residents’ knowledge. This reflects the similar and wider situation at the national level. A recent study conducted on a sample of Italian hospitals revealed that not every assessed hospital had a formal training program (e.g., drills, simulations, cross-training in high-demand services) for health-care providers [44]. Promoting and enhancing the training capacity in the field of disaster medicine is one of the “call-to-action requirements” requested by the international community [45]. Teams of both editions of the competition also scored poorly at the obstetrical emergency station, particularly Ed 1. This is no surprise, as obstetrical emergencies are known to commonly induce intense stress [46].

Nowadays, increased alertness is directed towards non-technical skills as an essential component of optimal management of emergencies [47]. Our findings regarding team performance suggests that teams who are very strong in non-technical skills also appear to be very strong in technical skills, and the same may be true for very weak teams. A similar correlation has been shown in previous research [48, 49]. Conversely, teams in the mid-scale have much more variable technical abilities. This finding may also suggest that in this setting, simulation performance requires assessment of both technical and non-technical skills.

## Limitations

There are several limitations to this study. Due to the practicalities of event, registration teams had heterogeneous post-graduate training experience and skill-set; however, this also might happen in real life. Some results rely on the self-assessment of the residents who were queried, which is not always an accurate indicator of actual competence. Whether the increase in self-confidence that residents report actually translates to improved performance in clinical medicine and subsequent patient outcomes is beyond the scope of this study and would be an interesting follow-up study. Moreover, a participant selection bias may apply as residents who do not thrive to participate in such events might not achieve the same results and might not find this educational format suitable to their needs.

Second, it remains to be seen if these results can be generalized to other medical specialties and settings beyond the ones in the present study.

Finally, it is important to recognize that the SIMCUP is a very intensive but brief learning experience. As such, the educational benefits to participants may be limited by this short encounter time.

## Conclusion

This study suggests that simulation competition may serve as a relevant instructional format in residency training. Residents were able to practice their skills and demonstrate abilities in a competitive yet controlled environment while experiencing and developing clinical management skills, psychomotor abilities, communication strategies, and teamwork skills. The performance assessment method allowed delineation of both the clinical areas and the skills, in terms of technical and non-technical, which deserve a corrective intervention.

The surveyed residents reported high satisfaction with simulation experience and a greater improvement of self-perceived competency and proficiency.

## Additional file

Additional file 1: Self-perceived confidence results for each station before and after each event (10-item Likert scale). Results are median and 25–75 percentile. Performance assessment score for each station regarding both skill scores and non-technical skills––global rating scale (GRS). Skill scores range from 0 to 1 while GRS from 0 to 42. Results are median and 25–75 percentile. Relationship between technical skill score (at in this chart) and non-technical skill score GRS (overall in this chart) for each team in each station. (DOCX 29 kb)
