# Supplementary material for: A novel simulation competition format as an effective instructional tool in post-graduate medical education
Source: Adv Simul (Lond). 2018 Aug 9;3:17. doi: 10.1186/s41077-018-0075-4 (PMC6085625; doi:10.1186/s41077-018-0075-4)

Additional file 1

Self-perceived confidence results for each station before and after each event (10-items likert scale). Results are median and 25-75 percentile.

|  | **Ed.1** | | | **Ed.2** | | |
| --- | --- | --- | --- | --- | --- | --- |
|  | **PRE** | **POST** | **p** | **PRE** | **POST** | **p** |
| **High Fidelity, Medical Adult (1)** | 6 [4-7] | 7 [6-8] | 0.012 | 6 [4-8] | 8 [7-9] | 0.035 |
| **Medium Fidelity, ACLS (2)** | 6 [4-7] | 8 [7-9] | 0.001 | 6 [4-8] | 8 [7-9] | 0.001 |
| **Virtual Reality, Disaster Triage (3)** | 3 [2-3.75] | 4 [2.75-5] | 0.004 | 5 [3-5] | 5 [3-7] | 0.036 |
| **High Fidelity, Pediatric/Newborn (4)** | 3 [2-4] | 4.5 [3-6] | 0.002 | 3.5 [2.25-5] | 5.5 [4-7] | 0.023 |
| **High Fidelity, Obstetrical Emergency (5)** | 3 [2-4] | 5 [3-6] | 0.003 | 4 [2.25-5] | 5 [4-7] | 0.039 |
| **High Fidelity, Prehospital Trauma (6)** | 4 [3-6] | 6.5 [5-8] | 0.005 | 5 [3.25-6] | 6.5 [5-8] | 0.001 |

Performance assessment score for each station regarding both Skill Scores and Non Technical Skills – Global Rating Scale (GRS). Skill scores range from 0 to 1 while GRS from 0 to 42. Results are median and 25-75 percentile.

|  | **Skill Score** | | **GRS** | |
| --- | --- | --- | --- | --- |
|  | **Ed.1** | **Ed.2** | **Ed.1** | **Ed.2** |
| **High Fidelity, Medical Adult (1)** | 0.93 [0.64-1.00] | 1.00 [1.00-1.00] | 35 [28-37] | 37 [30-40] |
| **Medium Fidelity, ACLS (2)** | 0.70 [0.30-0.90] | 0.69 [0.56-0.83] | 28 [14-28] | 22 [18-27] |
| **Virtual Reality, Disaster Triage (3)** | 0.20 [0.13-0.39] | 0.38 [0.19-0.75] | 7 [7-16] | 20 [18-27] |
| **High Fidelity, Pediatric/Newborn (4)** | 0.50 [0.50-0.66] | 0.69 [0.58-0.81] | 20 [15-32] | 18 [17-23] |
| **High Fidelity, Obstetrical Emergency (5)** | 0.36 [0.36-0.46] | 0.54 [0.50-0.75] | 20 [19-29] | 27 [20-31] |
| **High Fidelity, Prehospital Trauma (6)** | 0.79 [0.71-0.86] | 0.65 [0.50-0.90] | 26 [17-32] | 28 [18-30] |

Relationship between Technical Skills Score (at in this chart) and Non-Technical Skills Score GRS (overall in this chart) for each team in each station.


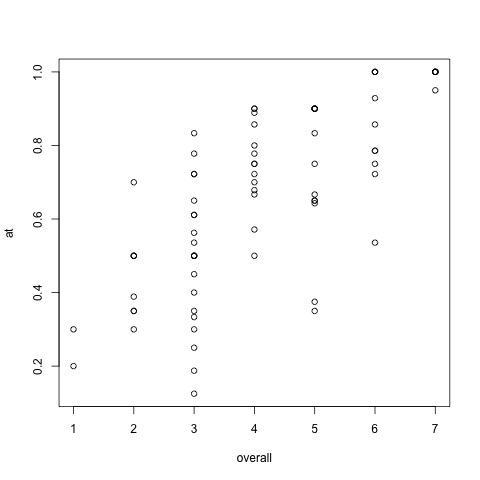

Supplement: Supplementary file 1 — Self-perceived confidence results for each station before and after each event (10-item Likert scale). Results are median and 25–75 percentile. Performance assessment score for each station regarding both skill scores and non-technical skills––global rating scale (GRS). Skill scores range from 0 to 1 while GRS from 0 to 42. Results are median and 25–75 percentile. Relationship between technical skill score (at in this chart) and non-technical skill score GRS (overall in this chart) for each team in each station. (DOCX 29 kb) [file 41077_2018_75_MOESM1_ESM.docx]
